# Gait as a vital sign: integrating wearables and AI into vestibular and balance medicine

**DOI:** 10.3389/fneur.2026.1736898

**Published:** 2026-02-24

**Authors:** Roger Kalla, Nicole Tiller, Mehul Goyal, Tatiana Brémovà-Ertl

**Affiliations:** 1 Department of Neurology, University Hospital Bern, Bern, Switzerland; 2 Department of Neuropediatrics and Center for Rare Diseases, University Hospital Inselspital, Bern, Switzerland

**Keywords:** artificial intelligence, balance, digital biomarkers, dizziness, gait, inertial measurement units, vestibular, wearable sensors

## Abstract

Safa Jabri, University of Michigan, United StatesGait and balance are integrative markers of brain–body function, reflecting the coordination of sensory precision, motor control, and cognition. Advances in wearable inertial sensors and artificial intelligence (AI) now enable continuous, objective measurement of these processes—positioning mobility assessment as a potential “ECG of the 21st century.” Dizziness and imbalance are among the most frequent primary-care complaints, affecting up to 30% of adults annually, while more than one-third of those aged ≥65 experience at least one fall each year. Digital gait metrics derived from wearable sensors quantify performance across the neuro-vestibular continuum, linking peripheral vestibular input, sensorimotor control, and higher cognitive function. Classical bedside tests such as the Romberg and Timed Up and Go have evolved into sensor-augmented paradigms that capture postural sway, gait symmetry, and head–trunk coordination with millisecond precision. Inertial measurement units detect vestibular and neurological impairments with fall-risk prediction accuracies of AUC 0.75–0.88. Rehabilitation approaches incorporating vibrotactile, proprioceptive, or virtual-reality feedback enhance balance and neuroplastic adaptation. Collectively, these developments define a paradigm shift: mobility becomes a digital vital sign. In the near future, patients may step onto intelligent floors or wear imperceptible sensors generating personalized “mobility fingerprints,” linking everyday movement patterns to neural integrity. Integration of wearable analytics and AI into clinical workflows may establish digital gait analysis as a cornerstone of preventive neurology and primary care.

## 1 Vision and conceptual framework—“the gait as a vital sign”

The capacity to stand and walk upright is among the most intricate achievements of human physiology. Each step is coordinated by millions of neural signals distributed across cortical, subcortical, vestibular, and proprioceptive networks. Because these systems operate as an integrated control hierarchy, mobility serves as a sensitive indicator of neural integrity. Subtle deviations in walking speed, stride symmetry, or variability often precede over neurological or vestibular symptoms, offering an early window into adaptability and cognitive function.

Over the past two decades, gait has been reframed as a measurable vital sign. Studenski et al. (1) first identified gait speed as the “sixth vital sign,” showing that walking pace predicts mortality and hospitalization as accurately as traditional physiological measures. Middleton et al. (2) extended this view, defining gait as a functional vital sign integrating cardiovascular, musculoskeletal, and neurological performance. Subsequent analyses demonstrated that stride-to-stride fluctuations—once dismissed as random noise—encode physiologically meaningful information about adaptability and neural control (3, 4). Within healthy ranges, variability reflects flexibility, but its loss signals rigidity and disease.

The miniaturization of inertial motion sensors (IMUs) and the widespread use of devices with built-in accelerometers have revolutionized how mobility is assessed. Movement can now be captured continuously and unobtrusively with millisecond precision. The concept of digital gait biomarkers (5) encompasses quantitative mobility metrics derived from wearable motion data, including spatial parameters such as step length and asymmetry, temporal features such as cadence and stride variability, and dynamic indices such as sway and angular velocity. In Parkinson’s disease and related neurodegenerative conditions, these measures often pick up disease progression earlier than traditional clinical ratings (6–8).

Evidence from systematic reviews underscores this transformation. In a comprehensive synthesis of 24 studies, Bradley et al. (4) found that wearable sensors consistently detect abnormalities predictive of future falls in Parkinson’s disease. The most discriminative parameters included gait and stride variability, trunk motion, walking speed, and stride length. Despite heterogeneity in study protocols and sensor placement, sensor-derived metrics reliably differentiated “fallers” from “non-fallers.” Importantly, combined models integrating both clinical and wearable metrics outperformed either domain alone. These findings underscore the promise of wearable sensors for detecting fall risk before incidents occur, though consistent definitions and measurement protocols are still needed. Taken together, the evidence indicates that digital gait analysis can offer both mechanistic understanding and clear clinical utility.

Artificial intelligence (AI) further expands this analytic landscape. Machine-learning models trained on wearable data can classify peripheral versus central vestibular dysfunction, identify disease phenotypes, and predict falls with accuracies approaching expert evaluation (AUC ≈ 0.75–0.88) (9, 10). These developments have catalyzed wearable neurology—the continuous quantification of brain–body interaction through motion analytics (11). Accordingly, this review primarily focuses on vestibular and balance-related disorders, while selected neurological conditions such as Parkinson’s disease and mild traumatic brain injury are included as illustrative examples where gait and balance assessment is clinically relevant.

Emerging studies reveal that each person expresses a distinct and reproducible gait signature reflecting their unique neuromechanical coordination patterns (12). These movement patterns remain consistent across different walking speeds and show linear changes related to balance, preferred pace, cadence, and step length. Using detailed motion analyses and machine-learning methods, individuals can often be identified with very high accuracy based only on their gait, suggesting that mobility reflects not just disease-related changes but also unique physiological traits. The relative stability of these patterns across situations supports their potential use for early detection, prognosis, and individualized rehabilitation.

Gait, balance, and dizziness can be viewed along a shared sensorimotor–cognitive spectrum. Vestibular signals define spatial orientation, proprioception helps steady movement, and cortical networks combine these inputs to produce coordinated locomotion. Disruption at any level—vestibular, cerebellar, or cortical—can lead to changes in gait or postural instability. Thinking about these functions as part of one continuum brings together otology, neurology, geriatrics, and rehabilitation, and highlights mobility as a common marker of brain–body interaction.

Much like the electrocardiogram made hidden cardiac rhythms measurable, digital gait analysis turns neuromotor control into a quantifiable signal. In future clinical settings, patients may stand on sensor-equipped floors or wear unobtrusive devices that capture gait and balance parameters, generating individualized mobility profiles for clinical interpretation. Artificial intelligence could then provide automated readouts of gait stability, symmetry, and variability—similar to how heart rate and blood pressure are reported today. If the ECG captures the electrical activity of the heart, gait may be viewed as reflecting integrated neural control of movement, serving as a potential digital marker of neural function and resilience (12).

## 2 Epidemiology and health burden

Dizziness, imbalance, and falls are among the most common and costly symptom clusters encountered in clinical practice. They can stem from vestibular, neurological, cardiovascular, or psychosomatic causes. Population studies consistently list dizziness as one of the three leading reasons for primary-care visits. In a landmark analysis of 1,000 internal-medicine patients, Kroenke and Mangelsdorff (13) reported that dizziness accounted for 3–5% of all visits—surpassed only by fatigue and headache—and that etiology was established in only 16% of cases despite extensive testing. Subsequent community data from the United Kingdom confirmed comparable frequencies: in a population-based postal survey of 2,474 adults, dizziness ranked among the most common self-reported symptoms, particularly in those with chronic conditions or reduced employment status (14). A systematic review of 31 primary-care studies by Bösner et al. (15) further refined these estimates, indicating that dizziness or vertigo accounts for 1–15% of consultations, with vestibular causes responsible for roughly 5–42% of cases. Across studies, 20–30% of older adults report chronic imbalance, and subjective improvement rates vary widely from 37 to 77%.

The public-health implications are considerable. Vertigo and dizziness generate nearly eight million ambulatory visits annually in the United States and contribute to 3–4% of emergency presentations. Falls related to balance disorders remain a leading cause of injury-related morbidity and mortality. Among adults aged ≥ 65 years, one in three experiences at least one fall each year; approximately 10% result in serious injury, and 5% lead to fractures or head trauma (16). Globally, falls claim about 684,000 lives and cause 172 million disabilities every year—more than transport injuries, drowning, and burns combined (17).

The economic burden mirrors this high prevalence. A systematic review of cost analyses estimated that vertigo and imbalance account for combined direct and indirect expenditures of roughly €70–80 billion annually across high-income countries (18). Each injurious fall incurs average medical costs exceeding US $10,000, largely due to hospital admissions, diagnostic imaging, and extended rehabilitation. Despite this impact, diagnostic accuracy in primary care remains limited: fewer than 20% of dizziness cases receive a specific etiologic diagnosis at the initial visit, and delays of 6 to 9 months are frequent (19).

Wearable motion sensors now offer a path toward quantification of this largely subjective symptom domain. Objective analysis of sway amplitude, step variability, and head–trunk coordination can detect instability and elevated fall risk before the first incident. Machine-learning models trained on inertial data achieve area-under-the-curve values of 0.80–0.88 for distinguishing fallers from non-fallers (20), transforming balance from a descriptive complaint into a measurable biomarker.

Demographic change amplifies the urgency. By 2050, the global population aged ≥ 60 years will nearly double (17). Reductions in habitual gait speed below 1.0 m/s confer a two- to three-fold increase in hospitalization and mortality risk (1, 2). Routine mobility screening in primary care—analogous to blood-pressure or pulse measurement—could enable early identification of individuals who would benefit from vestibular rehabilitation, balance training, or medication review. Telemetric monitoring supports follow-up after concussion, stroke, or inner-ear surgery, while aggregated gait data could evolve into a population-level “mobility index” for preventive health systems.

Implementation remains uneven. Barriers include limited clinician familiarity with digital metrics, lack of standardized thresholds, and incomplete reimbursement pathways. Most devices are validated under laboratory rather than real-world conditions, underscoring the need for multicentre trials in community and primary-care settings. Nevertheless, early pilot programs integrating wearable gait assessment into outpatient workflows have reduced diagnostic uncertainty by approximately 35% and improved targeted referral to vestibular therapy (19).

## 3 Clinical screening and classical tests

Clinical evaluation of balance and gait has traditionally relied on simple, inexpensive bedside tests that require little equipment yet capture key physiological features. Foundational among these is the Romberg test, introduced by Moritz Heinrich Romberg (21), which contrasts eyes-open and eyes-closed stance to probe proprioceptive and vestibular input. Variations such as tandem stance or the use of compliant (foam) surfaces increase task difficulty and help reveal how sensory weighting shifts under challenging conditions (22, 23). Quantitative sway metrics—including path length, velocity, and amplitude—were later incorporated to complement visual inspection and improve standardization (24).

The Fukuda or Unterberger stepping test measures angular deviation during marching in place with eyes closed. Although inter-individual variability limits its precision, it remains a practical screen for unilateral vestibular hypofunction, with greater deviation typically reflecting labyrinthine asymmetry. In patients with acoustic neuroma, it shows higher sensitivity than the Romberg test (25–28).

**Figure 1:**
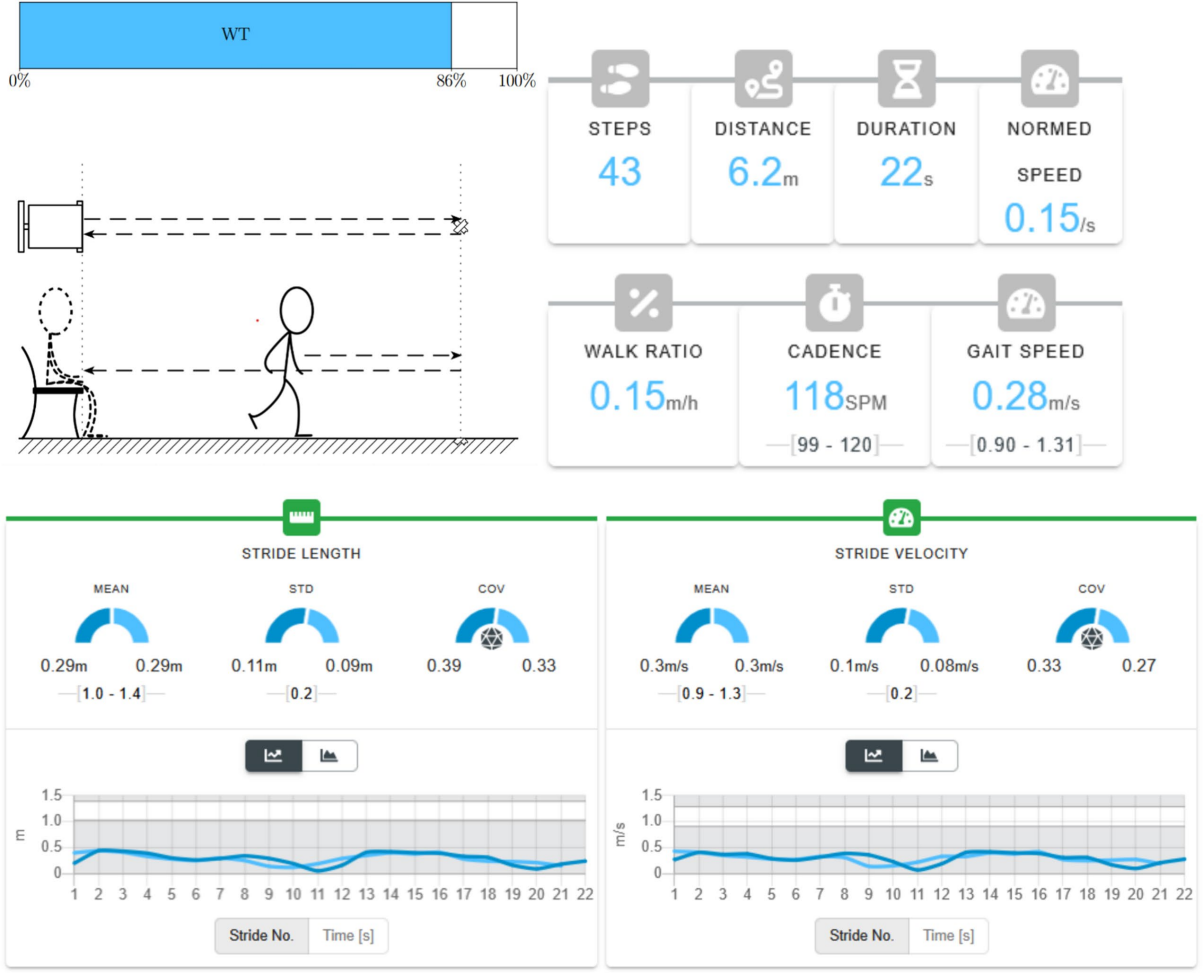
Timed up and go (TUG) test performance and gait parameters in an 81-year-old individual with Parkinson’s disease (90 kg, 182 cm).

The Timed Up and Go (TUG) test, developed by Podsiadlo and Richardson (29), combines sit-to-stand, straight walking, turning, and sitting into a single, functional task. Among older adults, completion times over 13.5 s are linked to roughly double the risk of falling. Its simplicity, reproducibility, and ecological validity make it a mainstay for mobility screening (Figure 1). Still, meta-analyses indicate that the TUG on its own has limited sensitivity for predicting future falls and should be used together with other complementary assessments (30).

The TUG test assesses functional mobility by measuring the time required to stand up from a seated position, walk 3 m, turn, return, and sit down. The participant completed the whole task in 25.24 s, with the walking phase accounting for 21.91 s (86.8%) of the total duration. The upper panel illustrates the proportion of walking time relative to total TUG duration, while the schematic below depicts the sequence of movements, including sit-to-stand, walking, turning, and return phases. During the walking phase, gait analysis recorded 43 steps over a 6.2 m distance in 22 s, corresponding to a gait speed of 0.28 m/s, cadence of 118 steps/min, and a walk ratio of 0.15 m/step/min. The normalized walking speed was 0.15 /s, indicating reduced gait velocity and rhythm typically associated with Parkinsonian bradykinesia and hypokinesia. These results reflect characteristic motor slowing and impaired mobility observed in Parkinson’s disease.

The lower panels display stride-by-stride metrics for stride length and stride velocity. Mean stride length was 0.29 m (SD 0.11 m, CoV 0.39), and mean stride velocity was 0.30 m/s (SD 0.08 m/s, CoV 0.27), both demonstrating high variability typical of Parkinsonian gait. These results reflect characteristic motor slowing and irregularity in stride generation. All measurements were obtained using the NuShu System.

The TUG shown in Figure 1 was recorded using a portable, sensorized shoe designed in a sneaker format, equipped with integrated inertial sensors and small actuators. This wearable setup enables medical-grade gait testing in everyday environments (31). Using built-in IMUs, pressure elements, and miniature force plates, it collects more than forty biomechanical parameters, including spatiotemporal measures such as gait speed, cadence, stride length, and stance time, as well as balance-related features like center-of-pressure path, symmetry, and sway. With AI-based analytics, systems such as the NuShu platform process these data streams in real time to produce individualized gait profiles and automated feedback. Machine-learning models trained on large normative datasets can describe movement quality and identify early pathological patterns. By combining classical bedside tests with embedded intelligence, such tools connect clinical tradition with digital technology, broadening access to quantitative mobility assessment.

The physiological principles underlying these simple tests help explain their effectiveness. Human stance control continually adjusts the weighting of visual, vestibular, and proprioceptive cues to maintain balance—a process that can be represented as adaptive feedback with context-dependent gains (32). Early perturbation studies demonstrated that postural responses are not rigid reflexes but learned, context-sensitive strategies (23, 24). This understanding justifies the diagnostic use of eyes-closed, foam-surface, and head-movement conditions and motivates newer dynamic versions of classical balance tests that probe multisensory integration (32).

Over the past decade, these traditional maneuvers have been extended into sensor-based protocols. Wearables and ambient sensors now yield millisecond-resolution kinematic data from smartphones, watches, trunk-mounted IMUs, and instrumented footwear (20, 31, 33). In community-dwelling older adults, IMU-derived gait metrics—speed, cadence, stride and step time, or TUG duration—show good to excellent validity compared with laboratory systems and strong test–retest reliability (34). Trunk accelerometry can reveal postural instability even in untreated Parkinson’s disease, detecting subtle sway features such as jerk or frequency dispersion that escape visual observation (33). Once classical tests are instrumented, categorical judgments like “normal” or “abnormal” become continuous multidimensional indicators of sway, symmetry, turning, and head–trunk coordination. Individual TUG phases can also be segmented automatically, correlating closely with instrumented gait variables (30, 34).

Large-scale screening with consumer devices is now technically possible, though accuracy in daily-life conditions still depends on sensor placement and sampling consistency (35). For example, instrumented shoes combining IMUs, pressure sensors, and micro-force plates can extract more than forty biomechanical features with minimal error relative to motion-capture systems, allowing real-time gait analysis beyond the lab (31).

Artificial intelligence further extends these capabilities. Machine-learning models based on wearable data can distinguish vestibular from non-vestibular gait patterns and stratify fall risk, though performance tends to be higher in controlled studies than in free-living environments (20, 35). Patient-reported tools such as the Vestibular Disorders Activities of Daily Living (VADL) scale provide complementary perspectives on functional limitation and participation (36). Together, clinician observation and wearable quantification offer a more complete view of mobility and balance function.

Several limitations remain. Performance is influenced by footwear, fatigue, attention, and environment; device calibration, sensor placement, and algorithm design affect reproducibility. Definitions of cadence, sway, and variability are not yet standardized across hardware platforms, complicating cross-study comparisons (34, 35). Nonetheless, hybrid workflows combining classical maneuvers (Romberg, Fukuda, TUG) with concurrent IMU recording provide a pragmatic bridge to clinical integration. Embedding digital outputs in electronic health records will allow longitudinal tracking alongside vital signs, while remote home-based assessments via smartphones or smart-shoe sensors could detect early mobility decline before patients present with dizziness or imbalance. As regulatory frameworks evolve, digitally enhanced balance testing is likely to achieve recognition as a validated digital biomarker pathway, comparable in status to ECG telemetry or continuous glucose monitoring (37–39).

## 4 Posturography and objective balance quantification

Posturography converts balance performance into continuous, multidimensional signals that reflect how vestibular, visual, and somatosensory inputs are integrated. Unlike categorical bedside tests, instrumented platforms and wearable systems quantify upright stance as trajectories of the body’s center of pressure (CoP) or overall sway. Traditional force plates capture key stability features—such as sway path length, mean velocity, root-mean-square amplitude, and 95% ellipse area—several of which can differentiate fallers from non-fallers with clinically relevant effect sizes. A recent meta-analysis identified sway area per unit time, anteroposterior mean velocity, and radial mean velocity as particularly informative CoP markers of fall risk in older adults (40).

Early studies using body-worn sensors demonstrated that trunk sway during stance tasks reliably distinguishes unilateral vestibular loss from healthy balance control, with the largest differences observed during eyes-closed foam stance, head-movement walking, and tandem walking on foam (41, 103). The reliability of posturographic measures is high across these paradigms (intraclass correlation coefficients generally above 0.85), supporting their use for longitudinal follow-up (42, 43). Epidemiological data underline the broader significance: in the NHANES 2001–2004 sample, 35% of U.S. adults aged 40 years or older showed vestibular dysfunction on a modified Romberg, and those with symptoms had roughly twelvefold higher odds of falling (44). Findings from large “living-lab” datasets echo this pattern, showing age-related decline in foam–eyes-closed performance and strong associations with diabetes and obesity; a time-to-failure under 10 s increased the odds of a recent fall by about fivefold (45).

Dynamic posturography extends static testing by introducing sensory conflicts—tilting surfaces, moving visual scenes, or compliant supports—to probe how the nervous system recalibrates balance when inputs disagree. This approach aligns with sensorimotor control models in which stability depends on feedback loops with task-specific gains—for example, greater vestibular weighting during larger perturbations—and helps explain why foam, eyes-closed, and head-movement paradigms are so diagnostically useful (41, 46).

Advances in wearable and mobile sensing have brought posturography beyond the lab. Trunk-mounted accelerometers and inertial measurement units (IMUs) can extract sway features such as jerk and spectral dispersion, detecting instability even in untreated Parkinson’s disease. These measurements agree well with force-plate outputs and are sensitive to subtle balance deficits (33). Across older populations, IMU-derived stance and gait variables show good to excellent validity and strong test–retest reliability, allowing balance assessment in community and home settings (43). Static posturography can also be implemented with low-cost hardware; derived cut-offs and Romberg Quotients from pressure-sensing boards can differentiate single from multiple fallers with reasonable accuracy, emphasizing the value of condition contrasts (eyes open vs. closed) and velocity-based metrics (47). Beyond scalar sway, head–trunk coordination provides another dimension: wearable cross-spectral analyses reveal restricted head motion, increased coupling, and reduced turning amplitude and velocity in vestibular hypofunction or post-schwannoma patients—abnormalities that may persist even after static sway normalizes (48). Full-body motion capture confirms similar changes in chronic vestibulopathy, where increased gait variability (GaitSD) and a shift from space- to trunk-centered head stabilization emerge as potential digital biomarkers (49).

Interpretation, however, requires clinical context. Greater sway is not always pathological—it can reflect exploratory adjustments or adaptive recalibration—while reduced sway may indicate rigidity, excessive visual dependence, or diminished postural flexibility. Therefore, results should be considered alongside clinical history, vestibular tests (e.g., vHIT, VOR gain), and functional outcomes. For example, patterns of compensatory saccades relate systematically to VOR gain reductions in healthy aging and may point to early vestibular decline that parallels postural changes (50). Patient-reported and functional measures add essential context on participation and perceived disability that instrumentation alone may overlook (51).

Methodological factors also shape data quality. Platform stiffness, stance width, trial length, sensor placement, sampling frequency, and filtering methods all influence CoP and sway metrics. Inconsistent definitions—for instance, of sway velocity or variability—further complicate comparisons across studies. Consensus efforts therefore emphasize standardized data collection, transparent processing, and linking of tests to a systems-based model of postural control that spans static and dynamic stability, anticipatory and reactive control, sensory integration, verticality, cognitive influence, and motor execution (52). Classic reviews echo this need for harmonized outcome sets and reproducible analysis pipelines to support clinical translation (46, 51).

Looking forward, posturography is merging with closed-loop rehabilitation. Real-time feedback—through sound, vibration, or mixed-reality cues—can reduce sway and improve balance in patients with vestibular loss, demonstrating that artificial sensory input can be integrated with residual function (53). These developments point toward home-based “balance telemetry,” where wearable sensors continuously stream stance and transition data to remote analytics that issue personalized feedback or fall alerts. As telehealth becomes routine, standardized, device-independent sway metrics validated against population norms (40, 44) and prospective outcomes (47) will be crucial to qualify posturography-derived features as regulatory-grade digital biomarkers for diagnosis, monitoring, and adaptive therapy.

## 5 Gait and functional mobility

### 5.1 Concept and quantification

Gait is a complex systems behavior that arises from the interaction of musculoskeletal strength, vestibular feedback, proprioception, and executive control. The use of wearable inertial measurement units (IMUs) has taken gait analysis beyond laboratory motion capture, allowing continuous, real-world measurement. Single- and multi-sensor systems can now estimate stride timing, step symmetry, walking speed, turning dynamics, and sub-phase events with good agreement to instrumented walkways and optical systems, making longitudinal monitoring feasible outside controlled settings (54).

In direct validation studies, accelerometer-based platforms capture a broad range of spatiotemporal features, showing excellent concordance for mean parameters and acceptable-to-good performance for variability and asymmetry measures—precisely the domains most relevant for instability (54). In early neurological cohorts, adding free-living mobility data to standard clinical and laboratory assessments improves prospective fall prediction, highlighting the added value of continuous, naturalistic monitoring (55).

Among scalar digital indicators, stride-to-stride variability remains one of the most sensitive markers of instability and future falls. Trunk accelerometry further provides frequency-domain and harmonic features that reflect vestibular–proprioceptive coherence (54, 56). Low-cost IMU platforms can differentiate vestibular hypofunction from healthy gait during simple walking tasks, suggesting a path toward scalable screening (57). Pharmacological responsiveness is also measurable: in Parkinson’s disease (PD), levodopa improves pace-related parameters and arm swing, while sometimes increasing quiet-stance sway—evidence of distinct neural pathways for gait and balance (58). Taken together, these metrics define gait as an integrated motor–sensory construct that can be sampled continuously in natural environments.

### 5.2 Clinical biomarkers and applications

Instrumented clinical tests form a bridge between traditional bedside assessments and quantitative biomechanics. The instrumented Timed Up and Go (iTUG) is a prime example, transforming a brief functional test into a multidimensional digital evaluation. By automatically segmenting the task into sit-to-stand, straight walking, turning, and sitting phases, iTUG reveals phase-specific impairments that total duration alone might conceal (59). In a review of 40 studies by Ortega-Bastidas et al., inertial sensors were the predominant technology, used in roughly three-quarters of cases, almost always combined with automated segmentation algorithms. Across populations, iTUG consistently distinguished healthy older adults from individuals with Parkinson’s disease, vestibular disorders, or frailty-related deficits, showing high reproducibility and adaptability across laboratory, community, and home settings (59).

Wearable modeling further extends this approach. Choi et al. developed an IMU-based prediction model capable of estimating TUG duration from normal walking data, achieving mean absolute errors below 1 s with only a single pelvis-mounted sensor (60). Such methods enable remote functional screening and continuous monitoring of mobility decline, particularly in older adults at risk for falls. Notably, TUG completion times above 13.5 s are associated with nearly double the fall risk, confirming its value as a quick and reproducible index of mobility safety.

In Parkinson’s disease, wearable sensors are particularly powerful for capturing movement beyond the clinic. Continuous monitoring quantifies daily motor fluctuations, detects freezing-of-gait (FoG) episodes, and correlates digital gait features with established scales such as the Unified Parkinson’s Disease Rating Scale (UPDRS) (61). Accelerometry provides individualized, ecologically valid data on gait and activity that complement episodic clinical evaluations. Building on this work, Zhang et al. trained machine-learning models on impaired pre-FoG gait patterns that could predict freezing events with over 80% accuracy and latency below 1 s—offering a basis for real-time cueing interventions (62). These studies illustrate the shift from descriptive to predictive digital gait analytics.

Subclinical motor fingerprints may even precede manifest PD. Features such as asymmetric arm swing, reduced axial rotation smoothness, and increased gait variability have been detected in non-manifesting LRRK2-G2019S carriers, suggesting early dysfunction in sensorimotor integration long before clinical onset (56). Such findings reinforce the potential of gait metrics as early biomarkers of neurodegenerative change.

Vestibular and balance disorders similarly benefit from wearable-based quantification. IMU-derived gait measures—including base-of-support width, mediolateral sway surrogates, and turning stability—can distinguish unilateral or bilateral vestibular hypofunction from healthy gait with high accuracy (57). In acute dizziness and vertigo, however, conventional tools such as the TUG, Functional Gait Assessment (FGA), or Gait and Truncal Ataxia Index (GTI) show limited power to separate central from peripheral causes unless demographic factors like age and sex are controlled (63). This underscores the need for combined wearable–clinical workflows to refine diagnostic precision in vestibular medicine.

Beyond diagnosis, digital gait analytics provide a quantitative way to track rehabilitation outcomes. In a randomized trial of 61 older adults with mild to moderate dementia, Schwenk et al. reported that a 3-month resistance and functional training program improved gait speed, cadence, stride length, and double-support time, with large effect sizes (Cohen’s d = 0.8–1.3) (64). Adherence exceeded 90%, and gains were greatest among participants with poorer baseline performance, illustrating both the feasibility and responsiveness of gait metrics—even in cognitively impaired populations.

Overall, wearable-augmented gait tests offer scalable, sensitive, and clinically meaningful tools. They enrich traditional assessments by quantifying the microstructure within gross performance, extend monitoring into daily life, and transform mobility from an occasional observation into a continuous, data-driven biomarker of neurological and vestibular health.

### 5.3 Continuous and translational perspectives

Increasing prospective evidence supports the clinical value of combining bedside evaluation with quantitative gait analysis and continuous, real-world mobility tracking. In a six-month prospective study including 333 patients with early neurological gait disorders—spanning vestibular, cerebellar, hypokinetic, vascular, and functional causes—and 63 healthy controls, Schniepp et al. integrated three complementary approaches: (i) multimodal clinical and functional fall-risk assessment, (ii) laboratory-based gait analysis, and (iii) 14-day inertial-sensor monitoring in daily life (55). During follow-up, 40% of patients reported at least one fall, and 21% sustained an injurious event. Multivariate regression models distinguished fallers from non-fallers and occasional from frequent fallers with accuracies of roughly 78 and 91%, respectively. While retrospective fall history remained the single strongest predictor, both instrumented gait parameters and free-living mobility features provided independent and complementary information—particularly for estimating the severity of subsequent falls.

These data point toward a pragmatic, stepwise workflow for clinical use:

(a) Use fall history for rapid first-line triage;
(b) In those identified as at risk, supplement with instrumented gait analysis and multi-day free-living monitoring to refine prediction of recurrent or injurious falls; and
(c) Individualize interventions—such as vestibular or balance rehabilitation, strength training, medication review, or cueing strategies—based on the sensor-derived risk profile.

The next major challenge is standardization. Global initiatives such as the Digital Mobility Outcomes (DMO) and Mobilise-D consortia are now working toward unified parameter definitions, acquisition protocols, and reporting conventions. Their goal is to clarify how digital mobility data can enhance diagnosis, improve risk prediction, support early detection of decline, and monitor treatment response—especially in conditions like Parkinson’s disease (65). As these consensus frameworks mature and reference values become widely adopted, regulatory agencies including the U.S. FDA and European Medicines Agency have begun recognizing digital mobility endpoints as exploratory or supportive biomarkers. This growing acceptance is laying the groundwork for their incorporation into clinical trials, rehabilitation programs, and ultimately, everyday neurological practice.

## 6 Vestibular-specific diagnostics using wearables

### 6.1 Concept and physiological foundations

Wearable sensors have opened new possibilities in vestibular assessment, turning complex balance physiology into continuous, quantifiable signals. In vestibular disorders, compensatory adjustments appear as altered head–trunk coordination, gait asymmetry, and changes in sensory weighting. By capturing these subtle patterns, wearable devices translate vestibular physiology into digital biomarkers of sensorimotor function.

The ISway protocol demonstrated that a single trunk-mounted inertial measurement unit (IMU) can reproduce center-of-pressure dynamics with high fidelity (intraclass correlation coefficients > 0.90) compared with laboratory force platforms (47). Similarly, an instrumented version of the Balance Error Scoring System (BESS) increased sensitivity to mild vestibular impairment by automating error detection and minimizing interrater variability (66). Dual-sensor configurations—typically mounted on the head and trunk—allow analysis of vestibulo-spinal coupling, where amplitude and phase relationships between head and trunk motion correlate with caloric weakness and dynamic visual acuity (49, 52). These methods extend classical vestibular tests into more naturalistic settings, capturing multisensory integration during real movement rather than in constrained laboratory tasks.

Turning and dynamic stability—two vestibular-dependent functions—are particularly sensitive to sensor-based analysis. In Parkinson’s disease, for instance, instrumented Timed Up and Go (iTUG) and turning metrics can reveal subtle deficits that standard scales such as the Berg or Tinetti fail to detect (66). Parameters like peak angular velocity and number of turning steps distinguish mild PD from controls (*p* < 0.001), illustrating how digital motion capture exposes impairments that often go unnoticed in conventional clinical testing.

### 6.2 Data-driven diagnostics and mobile platforms

Machine learning and mobile sensing technologies are now reshaping vestibular diagnostics. Jabri et al. (67) showed that supervised models—including Random Forest and deep-learning architectures—can separate vestibular gait patterns from age-matched controls with high accuracy (AUC ≈ 0.88), even when using a single IMU on the upper limb during eyes-closed walking. Feature-importance analysis highlighted reduced arm-swing amplitude as a key marker of vestibular hypofunction, pointing to upper-limb kinematics as an underexplored but highly sensitive aspect of vestibular compensation.

In parallel, Ahmadi et al. (68) applied advanced machine-learning models to distinguish central from peripheral acute vestibular syndromes in the EMVERT trial. Using video-oculography and posturography data, a geometric deep-learning classifier achieved an area under the curve (AUC) of 0.96—clearly outperforming traditional diagnostic scores such as HINTS (AUC = 0.71) and ABCD2 (AUC = 0.58). Notably, the algorithm’s feature weighting aligned with established clinical heuristics, improving interpretability and trustworthiness. These findings demonstrate that algorithmic pattern recognition can complement, rather than replace, expert judgment—especially in emergency triage where time-critical differentiation of central causes is vital.

Progress in portable and low-cost hardware has also expanded accessibility. Bohlke et al. (69) reviewed evidence showing that accelerometers, validated against optical motion capture and force-plate systems, provide accurate and scalable measures of standing balance across populations with aging, Parkinson’s disease, multiple sclerosis, and traumatic brain injury. Classification algorithms applied to these signals reliably distinguish fall-risk profiles, though adoption remains limited by inconsistent software standards and a lack of consensus on outcome metrics.

More recently, augmented- and mixed-reality systems have begun merging kinematic sensing with controlled visual perturbation. Margani et al. (70) validated a HoloLens 2–based Dynamic Gait Index in 26 healthy adults, integrating IMU and visual-tracking data. The headset correlated strongly with standard clinical scoring and captured detailed head- and eye-movement profiles, positioning AR platforms as promising tools for interactive vestibular diagnostics and rehabilitation.

Collectively, these advances redefine vestibular testing as a continuum that spans clinic, laboratory, and daily life—where wearables, mobile sensors, and AI algorithms jointly characterize balance control and its adaptive recalibration.

### 6.3 Toward integrated and continuous vestibular assessment

The line between vestibular diagnosis and therapy is becoming increasingly blurred. Feedback-driven training systems—using vibrotactile, visual, or proprioceptive cues derived from wearable data—can reduce sway amplitude by 20–40% after vestibular loss (71), promoting sensory reweighting and enhancing central compensation. Such closed-loop paradigms effectively turn assessment into treatment, transforming raw motion data into real-time rehabilitation feedback.

Despite rapid progress, key barriers remain. Most studies are still small, use heterogeneous protocols, and lack consistent sensor calibration. Technical issues such as magnetometer drift, sensor placement variability, and environmental interference continue to challenge reproducibility. Critically, there is still no large normative database linking wearable-derived vestibular metrics to gold-standard measures such as caloric irrigation, video head impulse testing (vHIT), or rotary-chair results.

Regulatory bodies—including the FDA and European Medicines Agency (EMA)—are now beginning to outline pathways for qualifying digital vestibular biomarkers. Their guidance emphasizes reproducibility, interpretability, and clinical validation. Once such standards are established, wearable-based vestibular telemetry could enable continuous home monitoring that unites diagnosis, rehabilitation, and outcome tracking within a single digital ecosystem—a framework where the inner ear meets intelligent sensing.

## 7 Therapy and rehabilitation

### 7.1 Principles and mechanisms of vestibular rehabilitation

Vestibular rehabilitation therapy (VRT) is an evidence-based, exercise-driven approach designed to promote central compensation through three main mechanisms: gaze stabilization, habituation, and balance retraining (72). By systematically challenging vestibulo-ocular and vestibulo-spinal pathways, VRT enhances sensory integration and supports neuroplasticity within brainstem and cerebellar circuits.

Classic work by Cohen et al. (72) showed that most recovery following unilateral vestibular loss—such as after acoustic neuroma resection—occurs within the first 3 weeks, driven largely by central rather than peripheral mechanisms. Tumor size affected residual deficits, but age and early postoperative therapy had little impact on the overall rate of adaptation, underscoring the brain’s inherent ability to recalibrate sensory input.

Modern wearable technologies now bring these principles into the digital era. Continuous monitoring of sway, head–trunk coordination, and gaze stability allows clinicians to tailor exercise intensity in real time. Adaptive algorithms can adjust stimulus complexity—modifying visual or proprioceptive challenges based on the patient’s ongoing performance—creating closed-loop rehabilitation systems that help sustain engagement and accelerate recovery.

### 7.2 Sensory feedback, virtual environments, and adaptive paradigms

Advances in sensory-augmentation technology have expanded VRT far beyond traditional clinic-based routines. Vibrotactile and proprioceptive feedback systems provide immediate cues about body orientation or sway, effectively substituting or reinforcing impaired vestibular input.

In a controlled experiment, Ma and Lee (73) demonstrated that a plantar-force–based vibrotactile feedback system reduced sway area by 36% and improved compensatory reactions to translational perturbations in healthy adults, illustrating the potential of somatosensory substitution to stabilize posture. Building on this concept, Bao et al. (74) ran an eight-week randomized, home-based trial in community-dwelling older adults using smartphone-linked vibrotactile trainers. Participants receiving sensory augmentation showed greater gains on the Sensory Organization Test (SOT) and Mini-BESTest than controls, confirming both the efficacy and feasibility of remote telerehabilitation with minimal supervision.

Proprioceptive focal stimulation is another emerging frontier. Devices such as Equistasi^®^ convert body heat into localized micro-vibration, amplifying proprioceptive feedback and improving postural control. In a crossover randomized trial, Romanato et al. (75) found that 8 weeks of Equistasi^®^ use enhanced ankle dorsiflexion and trunk kinematics, with corresponding changes in posturographic frequency bands related to vestibular function. A systematic review by Alashram et al. (76) supported these findings across five moderate- to high-quality studies, concluding that Equistasi^®^ significantly improves gait speed and postural stability in Parkinson’s disease while maintaining an excellent safety profile. Collectively, these results underscore the potential of multimodal sensory augmentation to improve balance in both neurodegenerative and vestibular disorders.

Parallel developments in virtual and augmented reality (VR/AR) have also broadened the scope of VRT. Immersive visual perturbations delivered through head-mounted displays promote habituation and multisensory recalibration, while see-through AR devices allow task-specific training in realistic settings. Soto-Varela et al. (77) reported that posturography-guided VR rehabilitation significantly improved composite balance scores in older adults after only five sessions—achieving outcomes comparable to ten-session protocols, thereby improving cost-effectiveness and adherence. Likewise, Krueger (78) showed that a user-worn see-through display projecting an artificial horizon mitigated motion sickness and reduced the number of required therapy sessions without compromising results. Together, these findings show how mixed-reality tools can optimize exposure, engagement, and dosing efficiency in vestibular rehabilitation.

**Figure 2:**
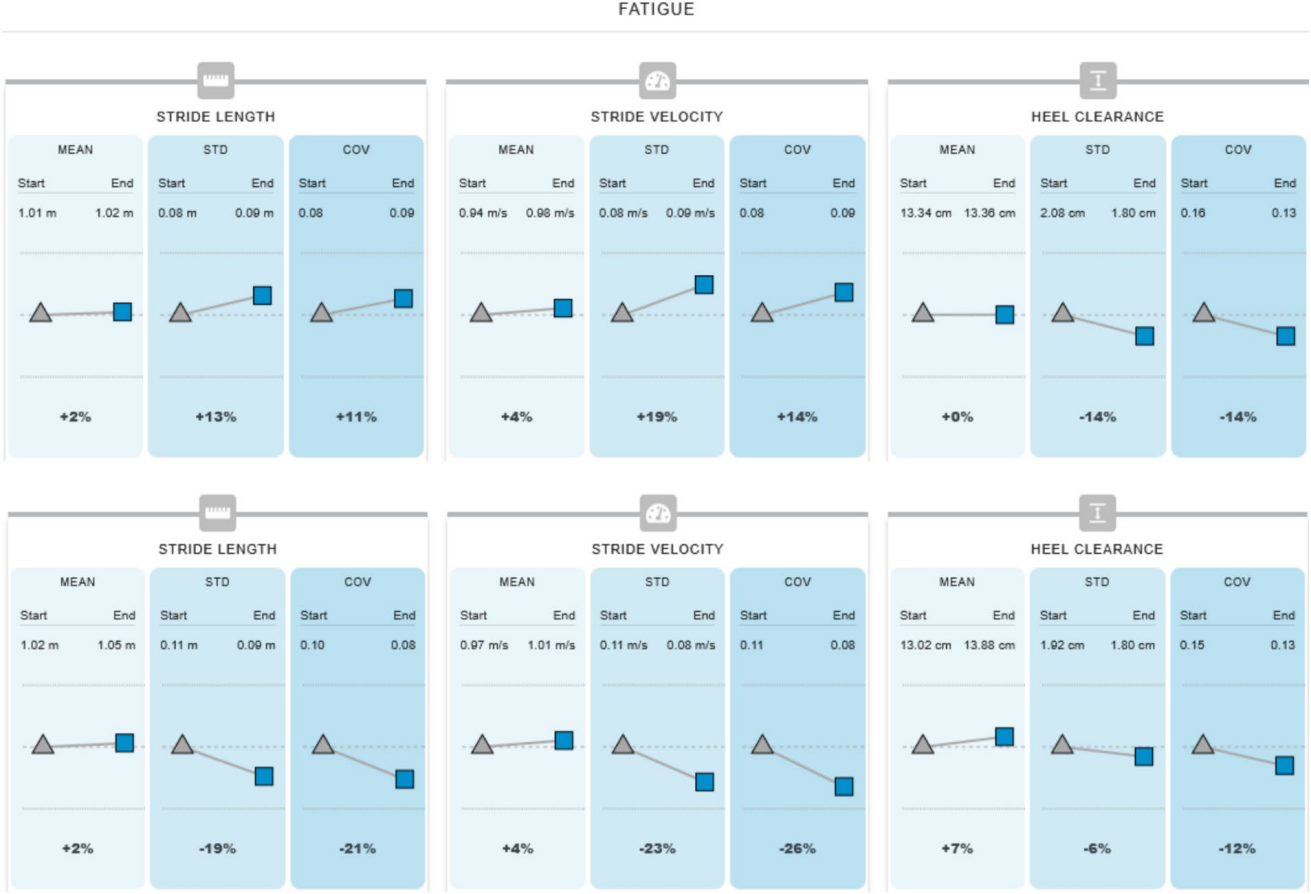
Gait performance and fatigue analysis in the same patient as Figure 3 during the six-minute walking test with and without vibration stimulation.

Figure 2 illustrates these principles in practice: an 88-year-old patient with bilateral vestibulopathy demonstrated improved walking performance and reduced gait variability when vibration stimulation was applied during a six-minute walk test. The intervention reduced stride variability (−21% COV in stride length; −26% in stride velocity) and mitigated fatigue-related instability, suggesting improved control and endurance under sensory-augmented conditions.

### 7.3 From episodic therapy to continuous adaptation

The convergence of wearable sensors, real-time feedback, and telemedicine is transforming vestibular rehabilitation from a series of scheduled sessions into a continuous, adaptive process. Lightweight sensors embedded in garments or insoles can now deliver subtle haptic or visual cues during daily activities—integrating therapy directly into walking, stair climbing, or other natural balance challenges.

Patients receive immediate feedback to correct instability, reinforcing motor learning in real-world contexts. Meanwhile, cloud-based dashboards aggregate mobility data, allowing clinicians to track recovery, identify performance plateaus, and adjust exercise programs remotely. This hybrid model blends therapy, prevention, and monitoring into a unified, intelligent workflow.

Ultimately, vestibular rehabilitation is moving toward a dynamic digital interface between patient and data—marked by continuous adaptation, sensor-guided personalization, and sustained engagement. In this emerging paradigm, the traditional boundaries between diagnosis, therapy, and daily function begin to fade, giving rise to a model of care where rehabilitation becomes continuous, contextual, and self-optimizing.

During the six-minute walking test without vibration, the participant took 656 steps and covered a distance of 330 meters in 5 min and 55 s. The normalized speed was 0.55 m/s, the gait speed averaged 0.93 m/s, the walk ratio was 0.55 m/h, and the cadence was 111 steps per minute. Under the vibration condition, the participant took 671 steps, covering 350 meters in 5 min and 58 s. The normalized speed increased to 0.58 m/s, with a gait speed of 0.98 m/s, a walk ratio of 0.56 m/h, and a cadence of 112 steps per minute. Overall, these results indicate slightly improved walking performance with vibration stimulation compared to the condition without vibration.

**Figure 3:**
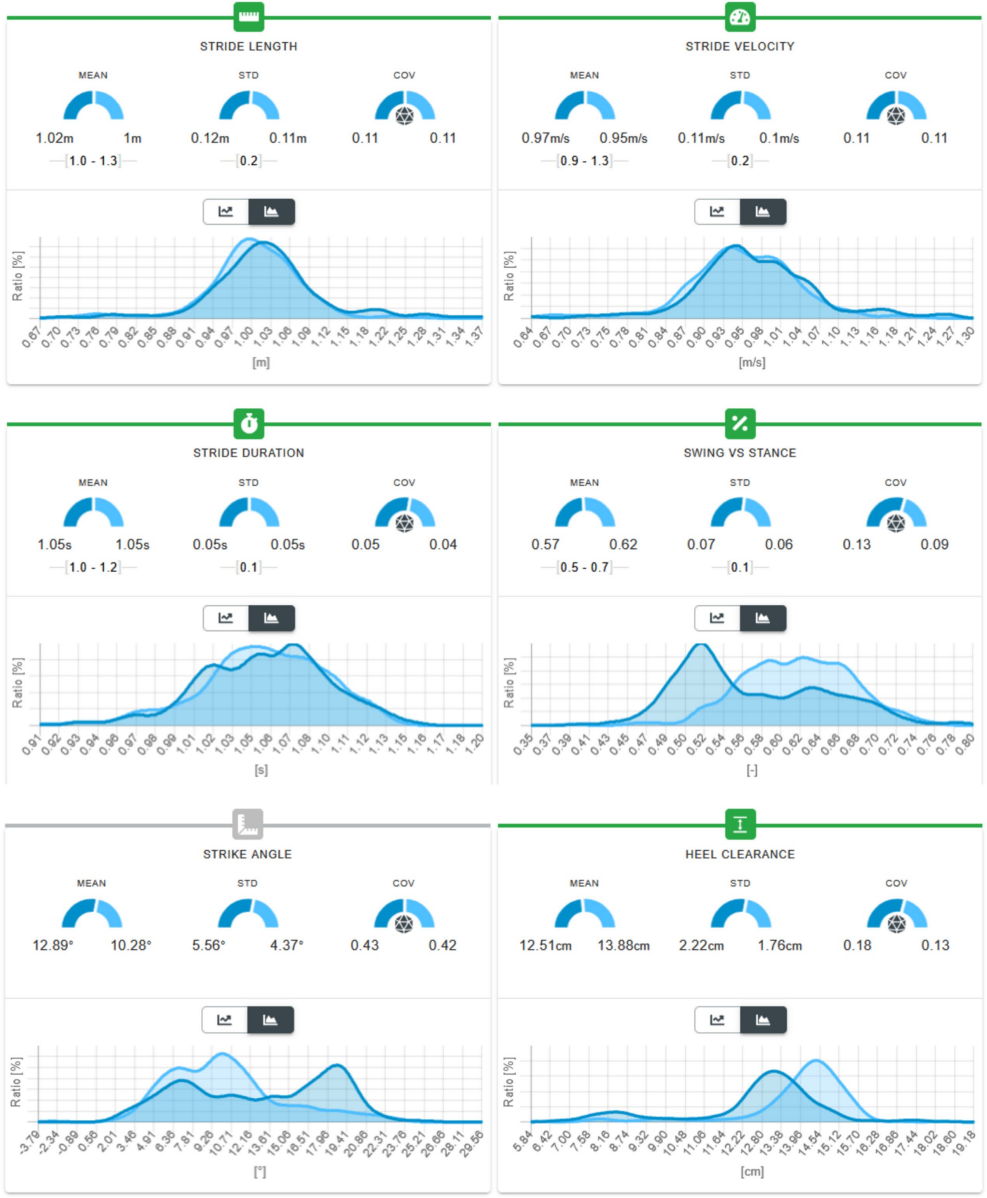
Six-minute walking test and gait parameters in an 88-year-old patient with bilateral vestibulopathy of unknown etiology. Upper row: The patient walked 330 m in 5 min 55 s, taking 656 steps, with a mean stride length of 1.02 m and a mean stride velocity of 0.97 m·s^−1^. The standard deviations (SD) for both parameters were low (0.12 m and 0.11 m·s^−1^, respectively), indicating a consistent and stable gait pattern throughout the test. Middle row: The mean stride duration was 1.05 s (SD 0.05 s), reflecting a steady rhythm and a balanced swing-to-stance ratio across strides. Lower row: Heel clearance measured 2.2 cm on the left and 1.76 cm on the right, suggesting mild asymmetry likely related to vestibular impairment. Overall, the gait pattern demonstrates reduced variability and moderate asymmetry, consistent with compensatory strategies often observed in chronic bilateral vestibulopathy.

Fatigue analysis in the same patient as Figure 3 illustrates the changes in mean, standard deviation (STD), and coefficient of variation (COV) for stride length, stride velocity, and heel clearance, comparing the first third and the last third of the six-minute walking test. Without vibration, the participant showed no significant change in mean stride parameters but exhibited an increase in variability. The mean stride length increased slightly by +2%, with STD rising by 13% and COV by 11%. Stride velocity increased by +4% in mean, with STD rising by 19% and COV by 14%, indicating greater inconsistency toward the end of the test. Heel clearance showed minimal change in mean but reduced variability (STD − 14%, COV − 14%). These findings suggest that although average gait parameters remained relatively stable, fatigue led to increased variability, reflecting reduced motor control and coordination.

In contrast, with vibration stimulation, the participant demonstrated improved gait consistency and reduced fatigue effects. The mean stride length increased slightly by +2%, while STD decreased by 19% and COV by 21%, indicating more consistent stepping. Similarly, stride velocity increased by +4% in mean, with STD decreasing by 23% and COV by 26%, reflecting enhanced stability. Heel clearance increased by +7% in mean, while variability decreased (STD − 6%, COV − 12%). Overall, vibration stimulation was associated with improved motor control, reduced gait variability, and better fatigue resistance during prolonged walking.

## 8 Disease-specific applications

The following sections highlight selected disease entities as illustrative examples, rather than providing a comprehensive comparison of treatment efficacy across conditions.

### 8.1 Parkinson’s disease

Wearable inertial measurement units (IMUs) have become central tools for phenotyping Parkinson’s disease (PD). They capture bradykinesia, stride asymmetry, freezing of gait (FoG), and postural instability with millisecond resolution, providing a digital complement to the UPDRS. In a large cross-sectional and longitudinal study, Schlachetzki et al. validated a multi-sensor, shoe-based system that quantified spatiotemporal gait in 190 PD patients and 101 controls, and sensitively tracked within-subject change over time—including stride length, stance time, and toe clearance (9). IMU-derived metrics captured the dopaminergic responsiveness of pace-related features while revealing increases in sway and postural instability that sometimes appear in the ON state but go under-recognized in conventional ratings. This demonstrates how wearable data can help separate therapeutic benefit from side effects.

Free-living monitoring further broadens the clinical reach of these systems. Validation studies consistently show that body-worn sensors yield reliable and clinically meaningful markers both inside and outside the clinic, linking digital parameters to PD severity and everyday function (54, 61). IMU-based analytics can detect FoG episodes and even identify prodromal gait changes that precede freezing, enabling predictions with lead times of about 0.9 s when models are trained on individually labeled pre-FoG periods (62). Medication effects remain domain-specific: levodopa reliably improves pace and arm swing but can also increase postural sway—especially in patients with dyskinesia—highlighting the value of multi-domain, sensor-derived metrics beyond simple gait speed (58). Together, these findings support routine, home-based motor monitoring as a means to individualize therapy and track disease progression over time.

### 8.2 Concussion and mild traumatic brain injury (mTBI)

Even after apparent recovery, subtle vestibulo-ocular and sensorimotor deficits often persist following concussion or mild traumatic brain injury. Gagné et al. found that a simple dual-task walking test performed in a corridor can expose lingering impairment roughly 10 weeks post-injury: while neuropsychological measures and symptom scores were comparable to controls, the mTBI group showed a significantly greater dual-task gait-speed cost and reported reduced concentration (79). These findings support the inclusion of cognitive-motor challenges to uncover residual dynamic instability.

Building on this approach, the START randomized trial protocol is evaluating whether earlier initiation of rehabilitation and wearable-assisted home programs improve outcomes—measured primarily by the Dizziness Handicap Inventory (DHI) and secondarily by postural and vestibulo-ocular metrics—with therapists remotely monitoring adherence and movement quality through sensors (80). Together, these methods point toward precision “return-to-play” and “return-to-duty” decisions grounded in objective mobility data rather than symptom reporting alone.

### 8.3 Persistent postural-perceptual dizziness (PPPD) and functional balance disorders

PPPD is characterized by chronic dizziness, visual dependence, and postural hypervigilance. Wearable systems can help quantify the underlying sensorimotor profile, such as increased mediolateral sway complexity or abnormal head–trunk coupling—signatures of maladaptive sensory reweighting. In a feasibility study involving patients with vestibular dysfunction, a digital vestibular rehabilitation platform combining a clinician dashboard, wearable sensors, and a patient app showed high usability (SUS ≈ 83) and significant reductions in dizziness, imbalance, oscillopsia, and anxiety after approximately 17 weeks of use, with strong adherence (81). Although larger trials are needed, these results illustrate how mobile vestibular technologies can operationalize PPPD as a disorder of sensorimotor prediction and provide continuous feedback to reshape maladaptive postural strategies.

### 8.4 Aging, cognitive decline, and neurodegeneration

Across aging and neurodegenerative conditions, gait speed and stride-to-stride variability remain among the most powerful predictors of morbidity, mortality, and functional decline. Foundational work by Hausdorff established that the variability and fractal dynamics of gait encode adaptability in locomotor control and deteriorate with disease (3). Both laboratory and free-living assessments link increased irregularity and rigidity of gait to executive dysfunction, mild cognitive impairment (MCI), and dementia risk. Importantly, targeted gait training can improve pace-related parameters even in cognitively vulnerable groups (64).

In differential diagnosis, IMU-derived gait signatures help distinguish ataxic (greater variability and mediolateral sway), hypokinetic (short steps, reduced arm swing), and vestibular (widened base, altered head–trunk coordination) phenotypes, allowing clinicians to tailor therapy accordingly.

Operational and resource-limited environments increasingly demand portable and durable assessment tools. A scoping review by Hoppes et al. identified several feasible technologies for field-ready vestibular assessment and rehabilitation, ranging from rapid screening and triage to remote monitoring. The review also outlined future directions—including AI-enhanced analytics, additive manufacturing, and implant-supported therapies—that could extend vestibular care well beyond tertiary centers (82).

## 9 Fall risk, prevention, and prognostic modelling

### 9.1 Epidemiology and clinical burden

Falls remain one of the most frequent, disabling, and costly geriatric syndromes. Around one in three adults aged 65 years or older experiences at least one fall per year, and roughly one in ten sustains a serious injury such as a fracture or head trauma. Beyond the immediate physical harm, falls accelerate functional decline, provoke fear of falling, and often precipitate institutionalization (83–87). Population-level data highlight the scale of this issue: in Canada, falls account for approximately 85% of all injury-related hospitalizations among seniors (85).

Prospective data confirm that falls are sentinel events marking broader physiological decline. Over 3 years, the risk of nursing-home admission rose progressively from one non-injurious fall (adjusted RR 3.1) to multiple non-injurious falls (RR 5.5) and to at least one injurious fall (RR 10.2) (87). Among community-dwelling fallers, nearly 70% reported some physical injury, and more than one-third experienced functional decline afterward. Predictors of poorer outcomes included female sex, higher medication burden, depressive symptoms, and falls occurring indoors (86).

Accurate risk profiling in acute-care settings remains a challenge. In older adults presenting to emergency departments after severe falls, self-reported diagnoses often disagree with general practitioner records—except for easily recognizable conditions such as Parkinson’s disease and diabetes. High concern about falling independently predicts this discrepancy, underscoring the need for interoperable health records and structured follow-up to guide secondary prevention (88).

Falls typically result from a convergence of intrinsic and extrinsic factors: vestibular dysfunction, sensory loss, muscle weakness, orthostatic hypotension, cognitive slowing, and polypharmacy on the one hand, and environmental hazards such as poor lighting, clutter, or improper footwear on the other (83, 84). Screening tools like the Timed Up and Go (TUG) test, Berg Balance Scale, and frailty indices offer useful stratification, though their individual-level predictive accuracy remains moderate (AUC ~ 0.70–0.75) (89).

### 9.2 Wearables and predictive modelling

A paradigm shift toward continuous, real-world fall prediction is underway. Wearable sensors record fluctuations in gait and balance across days or weeks, revealing control mechanisms invisible to brief clinical tests.

Enhancing standard tests with a single inertial sensor can markedly improve diagnostic precision. In nursing-home residents, for example, an iTUG setup using one lower-back IMU achieved 95.9% specificity and an overall accuracy of 74%, outperforming total TUG duration alone by incorporating angular velocity and turning dynamics (90). Similarly, accelerometry collected during ordinary walking can estimate TUG performance remotely with strong correlation (r ≈ 0.88), enabling clinicians to infer mobility status from natural activity data using a single pelvis-mounted sensor (60).

Free-living gait features also yield predictive insights. Dynamic stability metrics such as local dynamic stability (*λ*) differentiate recurrent fallers from non-fallers, with AUC values up to 0.75 depending on algorithmic parameters (91). Integrated models combining clinical assessment, laboratory gait analysis, and multi-day mobility monitoring improve six-month prediction of injurious falls, supporting a hierarchical workflow: first, fall history for rapid triage; second, instrumented gait for mechanistic phenotyping; and third, continuous telemetry for personalized intervention planning. Machine-learning approaches trained on multi-sensor data often reach AUC values near 0.90, confirming that digital gait signatures encode both motor control and postural stability mechanisms relevant to fall risk.

### 9.3 Interventions and preventive strategies

Falls are modifiable, and a growing body of meta-analytic evidence supports multifactorial prevention tailored to individual risk. Among community-dwelling older adults, structured exercise programs reduce the overall rate of falls by about 23% and the proportion of fallers by 15%. Balance and functional training cut fall rates by roughly 24%, multi-component interventions by 34%, and Tai Chi adds further benefit (92, 93). In institutional settings, vitamin D supplementation likely lowers fall rates in deficient groups, whereas evidence for medication review and broad multifactorial programs remains mixed, showing greatest effect in subacute hospital contexts (94).

Lifestyle-integrated interventions such as Stepping On or LiFE, which embed small balance and strength exercises into daily activities, reduce falls by around 31%, demonstrating the power of habit-based behavioral change (89). Perturbation-based balance training—targeting rapid, reactive postural responses—decreases both the proportion of fallers (RR 0.71) and overall fall rate (rate ratio 0.54), and recent reviews provide clear guidance on dosing and implementation (95, 96).

Medication optimization is another key strategy. Psychotropic and cardiovascular drugs represent leading fall-risk–increasing medications (FRIDs). The STOPPFall tool, developed across 14 medication classes, offers structured deprescribing guidance (97, 98). Evidence also indicates that psychotropic withdrawal and medication adjustment can significantly reduce fall incidence (93). Home safety modifications—particularly when led by occupational therapists—further decrease both rate and risk of falls, especially among high-risk individuals and those with visual impairment (93).

Wearable-assisted exercise and rehabilitation platforms are emerging as scalable complements. Sensor-based feedback training improves static balance, sway control, and selected gait variables with good feasibility and patient acceptance. However, larger and longer studies are needed to confirm durable benefits for habitual mobility and fall reduction (99).

Taken together, current data establish falls as both a preventable condition and a biomarker of broader health decline. Recurrent falls predict cognitive impairment, frailty progression, and hospitalization, reinforcing the need for continuous, data-driven fall management (86, 87, 99). By aligning wearable-based prediction with individualized intervention and adaptive follow-up, fall prevention is evolving from episodic screening into a dynamic, precision-oriented domain within geriatric medicine.

## 10 Future directions and regulatory outlook

### 10.1 From episodic assessment to continuous neuro-monitoring

The practice of balance and gait assessment is moving away from episodic testing toward continuous, unobtrusive neuro-monitoring. Miniaturized inertial measurement units (IMUs), millimeter-wave radar, and depth-sensing cameras can now record mobility throughout daily life, effectively turning living environments into passive laboratories. Artificial-intelligence models trained on these dense, real-world data streams already detect disease phenotypes, classify gait abnormalities, and predict falls with AUC values above 0.90 (10, 62).

Translating these advances into clinical practice remains challenging. Key obstacles include sensor drift, algorithmic bias, and the limited interpretability of black-box models. Reproducibility across devices, sampling rates, and environmental contexts is inconsistent, making cross-study comparisons difficult. To develop mobility biomarkers that generalize, future research will need to prioritize explainable AI, transparent validation pipelines, and harmonized multicenter datasets. Only through such methodological discipline can digital gait analytics achieve the reliability needed for regulatory and clinical use. Beyond validation and regulatory challenges, the widespread clinical adoption of wearable-based gait and balance assessment is also limited by practical factors, including the lack of large-scale longitudinal outcome studies, clinician training requirements, reimbursement pathways, and issues related to patient acceptance and passive monitoring in daily life.

### 10.2 Standardization, regulation, and clinical translation

Recognizing these challenges, international initiatives such as Mobilise-D and the Digital Medicine Society (DiMe) have established frameworks for digital mobility outcomes, emphasizing sensor calibration, algorithm documentation, and interoperability (52, 100, 101). The Mobilise-D consortium—comprising 34 partners across 13 countries—has launched a large, longitudinal validation study of real-world mobility metrics in neurological, respiratory, and geriatric populations to link wearable-derived data with clinical endpoints and regulatory requirements (100). In parallel, consensus statements have defined core constructs such as walking, turning, and real-world walking bouts to harmonize data acquisition and reporting across centers (101).

Regulatory bodies are now following suit. The U.S. Food and Drug Administration (FDA) and the European Medicines Agency (EMA) are both developing qualification pathways for digital mobility endpoints. The FDA’s 2023 guidance on Digital Health Technologies for Remote Data Acquisition in Clinical Investigations (102) identifies walking speed, cadence, and step count as leading candidates for regulatory-grade biomarkers. This shift signals a pivotal moment: mobility data are evolving from exploratory research measures into clinically actionable endpoints suitable for therapeutic monitoring, risk stratification, and clinical-trial design.

As analytic pipelines mature, integration with electronic health records (EHRs) will enable automated alerts for instability, deconditioning, or early neurodegenerative change. On a population scale, aggregated mobility databases could inform public-health surveillance, guide resource allocation, and shape the design of age-friendly communities. The convergence of clinical and real-world mobility data thus establishes the foundation for a digital ecosystem that links preventive neurology, rehabilitation, and geriatric medicine.

### 10.3 The smart clinic: a conceptual outlook

In a conceptual “Smart Clinic,” mobility could be measured as routinely as blood pressure. Patients might step onto sensor-embedded floors or wear lightweight, instrumented footwear during standard exams. Motion data capturing gait symmetry, postural sway, and reactive balance would stream directly into the electronic chart, analyzed in real time by validated AI algorithms to generate interpretable feedback for clinicians.

Each patient’s mobility fingerprint could then appear in the EHR, visualizing trends in gait speed, sway patterns, and fall risk. On the population level, aggregated metrics could contribute to a mobility index analogous to cardiovascular or metabolic indices, providing dynamic surveillance of functional health across societies.

This vision reframes care from episodic evaluation to continuous adaptation, grounded in objective, real-world physiology. Rather than replacing clinicians, the Smart Clinic would augment their judgment—turning every encounter into a node within a larger network of precision monitoring that connects individual movement to population-scale insight. Within a conceptual and illustrative framework.

## 11 Conclusion: toward a digital physiology of balance and gait

Balance and gait are no longer peripheral clinical observations. They represent integrated expressions of brain–body communication—dynamic signatures of sensory precision, motor coordination, and cognitive control. From the bedside assessments of Romberg and Unterberger to today’s AI-enabled wearables, our understanding of equilibrium has evolved from qualitative description to quantitative neurophysiology.

Modern digital technologies now capture the subtle rhythms of walking, swaying, and turning as neural vital signs that reveal both vulnerability and adaptability. They quantify not only the consequences of disease but also the latent resilience of the sensorimotor system—the capacity to remain upright, reactive, and mobile in a constantly changing world.

As miniaturized sensors, artificial intelligence, and telemedicine continue to converge, mobility is emerging as a universal biomarker of healthspan, linking preventive neurology, otology, and geriatrics. These tools support early detection of subclinical decline, objective tracking of rehabilitation, and population-level analytics that are redefining how functional health is measured.

The conceptual “Smart Clinic” embodies this transformation: an intelligent clinical environment equipped with embedded sensors and depth-sensing arrays that continuously capture balance and gait parameters during routine encounters. Real-time dashboards could display spatiotemporal metrics, stability indices, and fall-risk probabilities, allowing clinicians to interpret movement as readily as vital signs. The examination room itself could function as a living laboratory for preventive neurology and precision geriatric care—where assessment, feedback, and intervention merge into a single adaptive process.

Ultimately, the next decade will not simply digitize movement—it will redefine it. Mobility will become a living physiological interface between the human body and its data. In this emerging paradigm, gait as a vital sign is no longer a metaphor but a clinical reality: a cornerstone of digital neuroscience and a foundation for future healthcare devoted to preserving autonomy, stability, and quality of life across the lifespan.
